# Self-care practice among adult hypertensive patients at ambulatory clinic of tertiary teaching Hospital in Ethiopia: a cross-sectional study

**DOI:** 10.1186/s40545-022-00421-3

**Published:** 2022-03-22

**Authors:** Tsegaye Melaku, Bodena Bayisa, Helen Fekeremaryam, Alemayehu Feyissa, Alemayehu Gutasa

**Affiliations:** 1 Department of Clinical Pharmacy, School of Pharmacy, Institute of Health, Jimma University, Jimma, Ethiopia; 2 Center for Disease Control and Prevention, Oromia Regional Health Bureau, Finfinnee, Ethiopia; 3 Department of Pharmacy, Black Lion Specialized Hospital, Addis Ababa University, Addis Ababa, Ethiopia

**Keywords:** Blood pressure control, Hypertension, Self-care practice, Self-efficacy

## Abstract

**Background:**

Globally, hypertension is a major public health problem and a strong predictor of cardiovascular disease complications. To reduce and prevent complications from hypertension, it is important to adapt self-care behaviors. This study aimed to assess the self-care practices of adult hypertensive patients at a tertiary teaching hospital in Ethiopia.

**Methods:**

A health facility-based prospective cross-sectional study was conducted at Jimma Medical Center in Ethiopia between February 2021 and July 2021. Binary and logistic regression was performed to assess the strength of associations between independent and dependent variables. Data entry and analysis were done using Statistical Package for social science (SPSS) software version 22.0. A *p*-value < 0.05 was considered to declare statistical significance.

**Results:**

From a total of 422 respondents included to the study, male accounted 55.7% and the mean ± SD age of the respondents was 58.7 ± 9.75 years. About 53.1% of patients had poor self-care practices toward hypertension. Not attending formal education [AOR = 2.15; 95% CI (1.74, 6.39); *p* ≤ 0.001], uncontrolled blood pressure [AOR = 2.14 95% CI (1.27, 3.61); *p* = 0.003], chronic disease co-morbidity [AOR = 1.48; 95% CI (0.25, 7.73); *p* ≤ 0.001], unfavorable attitude toward hypertension[AOR = 3.13; 95% CI (1.95, 7.52); *p* ≤ 0.001], and poor social support [AOR = 2.75; 95% CI (1.45, 6.43); *p* ≤ 0.001] were independent predictors of poor self-care practice.

**Conclusion:**

The level of self-care practices for hypertension in the study area was low. In particular, the level of adherence to the DASH diet, exercise, and weight control was very low. Patient-specific targeted interventions are required to improve self-care practices for hypertension.

## Background

Hypertension (HTN) is a growing problem affecting approximately one billion people worldwide, two-thirds of them in low-income countries [1]. Alarmingly, 1.56 billion adults are estimated to have HTN in 2025 [1, 2]. HTN is a leading risk factor for cardiovascular disease (CVD) for stroke, myocardial infarction, congestive heart failure, decline, and death [1, 3, 4]. HTN is a major risk factor for CVD, causing 45% of cardiovascular morbidity and mortality worldwide [2]. In most cases, this condition remains asymptomatic until followed by a stroke, myocardial infarction, kidney failure, or vision problems [1, 5, 6].

In Ethiopia, hypertension is the most common non-communicable disease (NCD) with a prevalence rate of 19.6% [7, 8]. Uncontrolled high blood pressure can lead to heart attack and eventually heart failure and stroke, kidney failure, blindness, blood vessel rupture, and cognitive impairment [9]. HTN complications are estimated to cause about 9.4 million deaths each year, representing 17% of all deaths worldwide [10, 11]. The main cause of poor control of HTN is the inability to comply with HTN’s self-care practice [12, 13]. Self-care practices have been an important and cost-effective tool in the management and prevention of hypertension and its complications. Self-care of hypertension includes adherence to medication, intake of a low-fat diet, daily exercise, restriction of alcohol intake, smoking cessation, weight loss, self-monitoring of blood pressure (BP), regular health checkups, and reducing stress [14, 15].

Lifestyle changes, formerly known as non-drug therapies, play an important role in people with and without hypertension. It may serve as an early intervention before initiating medication in people with high blood pressure and maybe an adjunct to medication for those already on medication [16, 17]. Lifestyle changes can lower systolic blood pressure by about 4–11 mmHg. It is also estimated that for every 1 kg weight loss, blood pressure decreases by 1 mmHg [9, 17].

Although high blood pressure can be modified and treated, there is little knowledge about the treatment of hypertension in developing countries. In developing countries, more than three fourth of the burden of hypertension is attributable to a lack of knowledge and inadequate practice of self-care measures [10, 18, 19]. In previous studies conducted in Ethiopia, the prevalence of inadequate self-care practices reaches 77% [20, 21], which is very high as compared to the World Health Organization (WHO) recommendation.

Several factors have been associated with adherence to self-care activities, including socio-economic status, age, gender, educational status, place of residence, co-morbidity, access to health care, level of health literacy, length of therapy, culture, social support, self-efficacy, source of information on self-care and knowledge of disease and treatment [22–26].

Adhering to self-management practices in hypertension is essential for patient management to achieve desired treatment goals by improving quality of life, preventing complications, and reducing health care costs, but self-care practices remain low in developing countries [26–28]. Measuring the level of self-care activity for hypertension, along with determinants, is important for establishing a successful strategy for the treatment of hypertension. The literature recommends different self-care practice evaluation methods. One of the validated assessment scales routinely used is Hypertension Self-Care Activity Level Effects (H-SCALE) [29]. It addresses most of the self-care behaviors of the patients which are expected to affect glycemic control. There are studies in Ethiopia on HTN self-care practices. However, the level of hypertension self-care practices and contributing factors have not been well studied in the southwestern part of Ethiopia. Therefore, the study aimed to evaluate the self-care practices of adult hypertensive patients in an outpatient clinic of a tertiary teaching Hospital in Ethiopia.

## Methods

### Study setting

The current project was conducted at Jimma Medical Center in Ethiopia. Jimma Medical Center (JMC) is one of the oldest public hospitals in the country. Geographically, it is located in Jimma city 352 km southwest of Addis Ababa, the capital. With a bed size of 800, JMC is the only teaching and tertiary hospital in the southwestern part of the country. It provides services for approximately 15,000 inpatients, 160,000 outpatient attendants, 11,000 emergency cases, and 4500 deliveries per year coming to the hospital from the catchment population of about 20 million people. The medical center has over 1500 health workers. Besides serving as a referral center, the hospital serves as a teaching and research center for several health professionals at the undergraduate and postgraduate levels. The hospital has different general departments and specialty units like surgery, pediatrics and child health, internal medicine, oncology, gynecology-obstetrics, ophthalmology, dentistry, and other subspecialty clinics. The hospital provides screening and treatment services for people living with chronic disease at different specific ambulatory clinics.

### Study design and period

A hospital-based cross-sectional study was conducted from February 2021 to July 2021.

### Study population

The study population for the current study was all adult hypertensive patients on follow-up at the ambulatory clinic of JMC during the study period and fulfills the inclusion criteria.

### Inclusion and exclusion criteria

All adult hypertensive patients aged 18 years and above on follow-up for greater than 3 months were included in the study. We excluded patients who were seriously ill and unable to respond to interviews during the data collection period.

### Sample size determination and sampling procedure

The sample size was determined using a single population proportion formula based on the following assumptions: considering 95% of confidence level, 5% margin of error, and 51% prevalence of poor self-care practice [30]. A minimum sample size was 384. Considering a 10% non-response rate, we recruited a total of 422 study participants consecutively over the study period. Participants were selected using a simple random sampling technique.

### Data collection tools and methods

Pretested structured questionnaires adapted from validated scales, published articles [30–34], and modified to the context of the study were used to collect data from participants. The tool contains socio-demographic information, clinical profile of patient, Self-efficacy measure, Social support, self-care practices, knowledge and attitude related information, and questions. Data were collected by the interviewer-administered questionnaire. Data were collected by three trained nurses with a Bachelor of Science degree. Blood pressure was considered when an average systolic BP > 130 and diastolic BP > 80 mmHg for at least three consecutive follow-up appointments in patients younger than 60 years old [35].

### Outcome measures and validating methods

**Table:** 

A	Self-efficacy	The patient’s self-efficacy is confidence in his or her ability to perform tasks. It is drawn from a five-item scale. Feedback options range from 0 (completely not confident) to 5 (completely confident). Respondents who scored 4 or higher were classified as having good self-efficacy, and if they scored below 4, it was considered poor self-efficacy [34]
B	Social support	Social support is when the patient has friends and others, including family, present in times of need or crisis to give them a bigger picture and a positive image of themselves. The Multidimensional Perceived Social Support Scale (MSPSS) was used to measure social support. The MSPSS is a 12-item measure that rates the adequacy of social support on a 7-point Likert-type scale ranging from 1 = strongly disagree to 7 = strongly agree. Total score from 12 to 84; with scores higher and equal to 64 indicates better (good) social support [36]
C	Self-care practice	Self-care practice was assessed using the impact of hypertension-related self-care activity level [29]. The H-SCALE is a self-report questionnaire that includes six categories of self-care behaviors, which include medication adherence, a low-salt diet, physical activity, smoking cessation, weight management, and abstinence from alcohol. Good self-care practices are considered when the patient scores average or higher on the H-SCALE questions
D	Medication adherence	Three items were used to evaluate adherence to the prescribed medication. Then add the answer to each item with a score range of 0 to 21. Participants scoring 21 points are considered adherent [37]
E	Blood pressure (BP) monitoring	Good blood pressure monitoring was considered when BP was measured one time per month and more than once per month for uncontrolled BP [38]
F	Diet quality	Diet quality was assessed based on the 11-item Dietary Approach to Stop Hypertension Quality (DASH-Q) scale. These factors allow us to evaluate healthy food intake related to the nutritional content of the DASH diet. Responses were summarized and ranged from 0 to 77. A score of fewer than 32 was considered poor diet quality. A score of 33–51 corresponds to the average quality of the diet and a score of 52 or higher corresponds to good diet quality [39]
G	Physical activity	Physical activity was assessed on two items and summarized responses on ranges from 0 to 14 score. Participants with a score of 8 or higher were considered to comply with the physical activity recommendations [40, 41]
H	Weight management	The patient’s weight management over the past 30 days was rated on a 10-item scale. Response categories range from strongly disagree [1] to strongly agree [5]. Summarize the responses to calculate a score in the 10–50 range. Participants reporting a score of 40 or higher were considered adherent to good weight management practices [33, 42]
I	Alcohol intake	The amount of alcohol consumed was evaluated on a 3-item scale. The participant who stated that they did not drink alcohol at all in the last 7 days, or generally did not drink alcohol at all, are considered abstainers [43]
J	Knowledge	Hypertension evaluation of lifestyle and management (HELM) scale was used to assess the knowledge of respondents. If a participant answers above the median knowledge question, it is considered “knowledgeable” or good knowledge [32]
K	Attitude	A “favorable attitude” is considered when a patient answers a question about attitude with an average or higher score

### Data processing and analysis

Data were entered into epi info and exported to the Statistical Package for Social Science (SPSS) version 22.0 for analysis. Descriptive statistics were used for the analysis of patient characteristics. Chi-square tests and logistic regression analysis were done to determine the presence of a statistically significant association between explanatory variables and the outcome variable. Factors associated with self-care practices were identified using bivariate and multivariable logistic regression analysis. Before analysis factors such as independence of errors, linearity in the logit for continuous variables, multicollinearity, and outliers were checked. Odds Ratio (OR), *p*-value, and their 95% Confidence Intervals (CI) were calculated, and the result was considered statistically significant at *p* < 0.05.

### Data quality management

The quality of the data was ensured through the development of appropriate data collection materials. Two-day training on study objectives, data retrieval, and the collection was given for the data collectors. Overall activity was monitored by the study's lead investigator.

### Ethical considerations

Ethical clearance was collected from the institutional review board of the institute of health, Jimma University. A permission and support letter were also obtained from the management committee of the hospital. Patients signed an informed consent form to participate in the study. The raw data were not made available to anyone and were not used as the determinant of the subjects. All steps in data collection and compilation were conducted and supervised by the principal investigator. Strict confidentiality was assured through anonymous recording and coding of questionnaires and placed in a safe place.

## Results

### Socio-demographic characteristics of participants

From a total of 422 respondents included in the study, males accounted for 55.7% and the mean ± SD age of the respondents was 58.7 ± 9.75 years. Nearly one-third (65.9%) of the respondents were married. About 33.9% of respondents had no formal education and 63% of the participants reside in an urban area. One-fifth of the participants had no regular income. About 278 (65.9%) of patients live with immediate family (Table 1).

**Table 1:** Socio-demographic characteristics of study participants at follow-up clinic

Variable	Category	Frequency	Percent
Age (years)	Mean ± SD	58.7 ± 9.75	
	18–35	30	7.1
	36–50	271	64.2
	> 51	121	28.7
Gender	Male	235	55.7
	Female	187	44.3
Educational status	Unable to read and write	143	33.9
	Primary school	119	28.2
	Secondary school	96	22.7
	College and above	64	15.2
Marital status	Single	42	10
	Married	278	65.9
	Divorced	31	7.3
	Widowed	71	16.8
Occupation	Gov’t/non-gov’t employee	89	21.1
	Self-employee	224	53.1
	Unemployed	109	25.8
Residence	Urban	266	63.0
	Rural	156	37.0
Average monthly Income (ETB)	No regular income	109	25.8
	≤ 3000	191	45.3
	> 3000	122	28.9
Living status	Living with immediate family	278	65.9
	Living with extended family	115	27.2
	Living alone	29	6.9

*ETB* Ethiopian Birr

### Clinical characteristics of participants

From the total of the included participants, more than one-fifth (26.8%) of them had had a family history of hypertension. About, 48.1% of participants had BMI above the recommended weight. About 249 (59%) of participants had uncontrolled hypertension. Nearly one-fifth (31.5%) of participants had chronic disease co-morbidity. About 114 (27%) of respondents missed their follow-up schedule (Table 2).

**Table 2:** Clinical characteristics of study participants at follow-up clinic

Variables	Category	Frequency	Percent
Family history of hypertension	Yes	113	26.8
	No	309	73.2
Body mass index	Underweight(< 18)	26	6.2
	Normal (18–24.99)	193	45.7
	Overweight (25–29.99)	173	41.0
	Obese(> 30)	30	7.1
Follow-up schedules	Every 2 weeks	16	3.8
	Every month	217	51.4
	Every 2 months	182	43.1
	Every 3 months	7	1.7
Blood pressure control status	Uncontrolled	249	59
	Controlled	173	41
Chronic disease co-morbidity	Yes	133	31.5
	No	289	68.5
Disease duration since diagnosis (years)	< 3	105	24.9
	3–5	101	23.9
	> 5	216	51.2
Treatment duration since diagnosis(years)	< 5	206	48.8
	≥ 5	216	51.2
Adherence to the follow-up schedule	Yes	308	73
	No**	114	27

**Missed at least one follow-up schedule within the last 3 months

### Therapeutic life change and self-care practice-related information

Of a total of included patients, 245 (58.1%) of them had good knowledge about self-care practice of hypertension. The majority (85.8%) of patients got information about self-care practice from a health care professional. About 282 (58.8%) of the participants had good knowledge about HTN self-care practice and 243 (50.6%) of the participants had a favorable attitude towards HTN self-care practice. More than half (51.9%) of participants had good social support and 259 (61.4%) had poor self-efficacy (Table 3).

**Table 3:** Therapeutic life change and self-care practice-related information of study participants at follow-up clinic

Variable	Category	Frequency	Percent
Knowledge towards self-care practice of hypertension	Good	245	58.1
	Poor	177	41.9
Source of information about self-care practice	Health care professional	363	85.8
	Relatives and friends	31	7.3
	Media	15	3.6
Attitude towards hypertension and self-care practice	Favorable	224	53.1
	Unfavorable	198	46.9
Self-efficacy	Good	259	61.4
	Poor	163	38.6
Social support	Good	219	51.9
	Poor	203	48.1

### Level of hypertension self-care practices

**Fig. 1:**
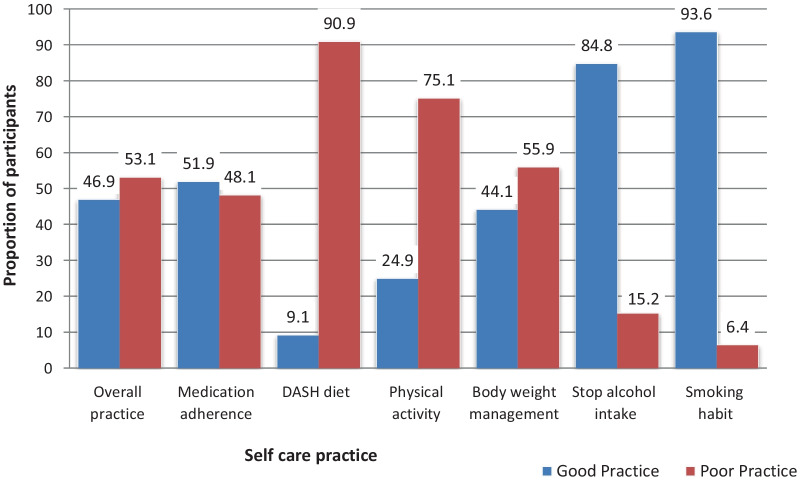
Level of self-care practice among study participants at follow-up clinic

The median score for self-care practice was 77 with a maximum score of 131. Out of the total respondents, 224 (53.1%) patients had overall poor self-care practice with 95% CI (49.37%, 57.13%). The median score for adherence to the prescribed medication was 19 with a maximum score of 21. About 203 (48.1%) of patients poorly adhered to their antihypertensive. The median score for dietary approach for stopping hypertension (DASH) practice was 17 with a maximum score of 55. Most of the participants 329 (78%) practiced low diet quality, 53 (12.6%) practiced medium diet quality and 40 (9.4%) practiced good diet quality. The median score for physical activity engagement was 6 with a maximum score of 12. About 317 (75.1%) respondents’ had a poor practice of the recommended physical activity. The median score for weight management practice among the participants was 36 with a maximum score of 48. More than half of respondents 236 (55.9%) had poor weight management practice. Few numbers 27 (6.4%) of participants were current smokers and 64 (15.2%) were alcoholics (Fig. 1).

### Factors associated with self-care practice

Bivariate analysis showed statistically significant associations between educational level, blood pressure control, disease co-morbidity, attitude to hypertension, adherence to follow-up schedule, and social support of the respondents. On multivariable logistic regression analysis revealed lack of formal education, disease co-morbidity, uncontrolled blood pressure, unfavorable attitude to hypertension, poor adherence to following up schedule, and poor social support were determinants for self-care practices. Patients who cannot read and write were two times more likely to practice poor self-care [AOR = 2.15; 95% CI (1.74,6.39); *p* ≤ 0.001] as compared to those who attended tertiary and above education. Hypertensive patients with uncontrolled BP were two times more likely to practice poor self-care [AOR = 2.14 95% CI (1.27, 3.61); *p* = 0.003] as compared to those who had controlled blood pressure. The probability of poor self-care was 1.48 times more likely among patients who had disease co-morbidity [AOR = 1.48; 95% CI (0.25, 7.73); *p* ≤ 0.001] as compared to those with no co-morbidity. Patients who had unfavorable attitudes were three times more likely to practice poor self-care [AOR = 3.13; 95% CI (1.95, 7.52); *p* ≤ 0.001] as compared to those patients with a favorable attitude. Additionally, hypertensive patients with poor social support were almost three times more likely to practice poor self-care practice [AOR = 2.75; 95% CI (1.45, 6.43); *p* ≤ 0.001] as compared to patients with good social support (Table 4).

**Table 4:** Factors associated with self-care practice of study participants at follow-up clinic

Variables		Self-care practice		COR (95% CI)	AOR (95% CI)	p-value
		Poor (n)	Good (n)			
Age (years)	18–35	12	18	1.00	1.00	
	36–50	141	130	1.85 (0.87, 7.68)*	1.34 (0.209, 3.33)	0.119
	> 51	71	50	1.25 (0.17, 5.22)*	1.07 (0.22, 8.31)	0.214
Gender	Male	123	112	1.00		
	Female	101	86	0.85 (0.41–10.46)		
Educational status	Unable to read and write	97	46	2.18 (0.73,13.38)**	2.15 (1.74, 6.39)	< 0.001
	Primary school	69	50	1.53 (0.92, 7.49)*	1.56 (0.81, 4.27)	0.136
	Secondary school	31	65	2.61 (1.15, 5. 85)*	1.24 (0.67, 5.21)	0.218
	College and above	27	37	1.00	1.00	
Residence	Urban	131	135	1.00		
	Rural	93	63	1.62 (0.48–7.35)		
Occupation	Gov’t/non-gov’t employee	41	46	1.00		
	Self-employee	113	111	0.47 (0.25–2.43)		
	Unemployed	70	39	1.31 (0.96–3.44)		
Living status	Living with immediate family	150	128	1.00		
	Living with extended family	65	50	0.94 (0.52–4.18)		
	Living alone	9	20	1.23 (0.70–3.91)		
Blood pressure control	Uncontrolled	107	66	4.26 (2.55, 11.34)**	2.14 (1.27, 3.61)	0.003
	Controlled	117	132	1.00	1.00	
Co-morbidity status	Yes	79	54	1.35 (0.43, 5. 83)**	1.48 (0.25, 7.73)	< 0.001
	No	145	144	1.00	1.00	
Adherence to follow-up	Yes	152	156	1.00	1.00	
	No	72	42	2.73 (0.98, 8.32)**	0.89(0.327,2.71)	0.067
Attitude to hypertension	Unfavorable	117	81	3.67 (2.26, 10. 98)**	3.13(1.95,7.52)	< 0.001
	Favorable	107	117	1.00	1.00	
Social support	Poor	121	82	3.17 (2.08, 11. 65)**	2.75 (1.45, 6.43)	< 0.001
	Good	103	116	1.00	1.00	

**p-value < 0.05; *p value < 0.25

## Discussion

Self-management is an important non-pharmacological approach that facilitates blood pressure control. It is essential for the prevention and treatment of hypertension [44]. This study aimed to determine the level of self-care in hypertensive patients in relation to antihypertensive drug compliance and therapeutic lifestyle changes. The overall level of self-care practice in this study was 53.1% (49.37%, 57.13%). This is consistent with a study in Nigeria [45] (47.4%) and a previous study conducted in a research setting [46] (55.3%). However, the result of this study was lower than the results of studies conducted in Addis Ababa [20] (77%), Durame [27] (72.7%), and MizanTepi [47]. Meanwhile, the result of this study was higher than those of studies conducted not only in Iran [48] (27%), but also in Harar [49] (37.9%) and Nekemte [50] (31.1%). These discrepancies may relate to differences in the population's lifestyle, cultural, economic situation, access to health care facilities, and educational attainment.

In this study, the rate of using a low-quality diet was 78% (69.66%, 84.47%). This result is higher than studies conducted in Durame [27], (42.5%) [27], Harar [49], (18.2%) [43], Saudi Arabia [45] (20.7%) [45] and Uganda [51] (24.4%). This may be due to differences in dietary perceptions on dietary management by region and country. In addition, differences in measurement tools, socio-economic and socio-cultural values may cause fluctuations in results.

In this study, non-adherence to physical activity was 75.1% (68.77%, 88.90%). This is consistent with a study done in Durame [27] (83.9%) and Saudi [52] (76.8%). However, this result was higher than the studies conducted in Addis Ababa [12, 53] (50.6% -68.6%) This may be due to limited facilities or the lack of a structural framework for exercise around the workplace as well as for the general public in the study area. In addition, patients' perceptions and attitudes toward physical activity may limit its practice.

The smoking rate in this study was 6.4%. This finding was lower than other studies done in Ethiopia [27, 49], India [54], Kenya [55] Nigeria [56, 57], Canada [58], Korea [59], and Iran [60]. In addition, the prevalence of alcohol use in this study was 15.2%. This was in line with other studies in Ethiopia [25, 27], Ghana [61], Kenya [55]. However, this result was much lower than studies conducted in other regions of Nigeria [56, 57] and Korea [59]. Differences in survey results may be related to differences in socio-cultural and religious viewpoints. Smoking and drinking within the community are not encouraged in this area of study.

Educational level showed a statistically significant relationship with self-management practice. Patients who cannot read and write were twice more likely to have poor self-care than patients with higher education. This was in line with the other studies done in Ethiopia [27, 62]. This may be due to cognitive differences between patients with formal education and those without formal education. Patients who cannot read and write overlook sources of information such as posters, flyers, and other written materials.

The probability of poor self-care practice was higher and statistically significant among patients with chronic disease co-morbidity. This finding was consistent with the other studies from Ethiopian settings [27, 53, 63]. This may be due to symptoms of co-morbidity that interfere with self-management of high blood pressure, or lifestyle changes caused by the co-morbidity. In addition, comorbid conditions exacerbate the patient's symptoms and prevent them from adapting to lifestyle changes [64].

In this study, people with uncontrolled blood pressure were twice as likely to be poorly self-managing as those with controlled blood pressure. This was in line with other studies [49, 62, 65, 66]. This may be due to the fact that good self-care habits can help control blood pressure.

This study also showed the impact of social support on self-management practices in patients with arterial hypertension. Patients with poor social support were nearly three times more likely to have poor self-management than patients with good social support. This was consistent with other studies [25, 53, 67]. This can be attributed to several aspects, of social support in stress management, and psychoactive self-help activities, that positively impact patients’ lifestyle changes. This study also showed a statistically significant relationship between attitudes toward hypertension and self-management practices. Patients with unfavorable were three times more likely to have poor self-care than patients with positive attitudes. This may be because hypertensive patients understand the complications of the disease and can engage in effective self-management to prevent these complications.

### Limitation of the study

As this is a one-hospital study, it will be difficult to generalize. Because the study participants’ self-management practices were based on self-report, there may be memory bias and social desirability bias.

## Conclusion

The level of self-care practices for hypertension in the study area was low. In particular, the level of adherence to the DASH diet, exercise, and weight control was very low. Not attending formal education, uncontrolled BP, poor social support, and unfavorable attitude, and living with chronic disease co-morbidity were independent predictors of poor self-care practices. Improving self-management practices for hypertension requires targeted, patient-specific interventions.

## Data Availability

All materials and data are available from the corresponding author without any restriction.

## Abbreviations

AOR: Adjusted odds ratio
CVD: Cardiovascular disease
COR: Crude odds ratio
CI: Confidence interval
BP: Blood pressure
DASH: Dietary approach to stop hypertension
DASH-Q: Dietary approach to stop hypertension quality
ETB: Ethiopian Birr
HTN: Hypertension
HELM: Hypertension evaluation of lifestyle and management
H-SCALE: Hypertension-related self-care activity level
JMC: Jimma Medical Center
MSPSS: Multidimensional Perceived Social Support Scale
NCD: Non-communicable disease
SPSS: Statistical package for social science
WHO: World Health Organization
